# Building a pipeline to solicit expert knowledge from the community to aid gene summary curation

**DOI:** 10.1093/database/baz152

**Published:** 2020-01-21

**Authors:** Giulia Antonazzo, Jose M Urbano, Steven J Marygold, Gillian H Millburn, Nicholas H Brown

**Affiliations:** Department of Physiology, Development and Neuroscience, University of Cambridge, Downing Street, Cambridge, CB2 3DY, UK

## Abstract

Brief summaries describing the function of each gene’s product(s) are of great value to the research community, especially when interpreting genome-wide studies that reveal changes to hundreds of genes. However, manually writing such summaries, even for a single species, is a daunting task; for example, the *Drosophila melanogaster* genome contains almost 14 000 protein-coding genes. One solution is to use computational methods to generate summaries, but this often fails to capture the key functions or express them eloquently. Here, we describe how we solicited help from the research community to generate manually written summaries of *D. melanogaster* gene function. Based on the data within the FlyBase database, we developed a computational pipeline to identify researchers who have worked extensively on each gene. We e-mailed these researchers to ask them to draft a brief summary of the main function(s) of the gene’s product, which we edited for consistency to produce a ‘gene snapshot’. This approach yielded 1800 gene snapshot submissions within a 3-month period. We discuss the general utility of this strategy for other databases that capture data from the research literature.

Database URL: https://flybase.org/

## Introduction

Biological databases have become essential resources for researchers by capturing, housing and displaying an up-to-date collation of data described in the primary research literature, including data on gene models and products, mutant alleles and their phenotypes, genand protein–protein interactions and gene product function. Unfortunately, the primary function of a gene product can often be obscured by the volume of data within each gene’s report page on the website, or worse, the key function may even be missing altogether. In these cases, even an experienced database user might need to spend a substantial amount of time analyzing numerous types of information to identify the most salient features of the gene. This task becomes even more arduous when faced with a large list of genes from a genome-wide RNA expression or proteomics experiment. Thus, a short summary of what is known about each gene and its products is very valuable to researchers.

Different databases have adopted different strategies for providing such a quick overview. Some have allocated curator effort to write summaries, whereas others compute them from data within the database. Manually written gene summaries are present in the Saccharomyces Genome Database (SGD) (1) and UniProtKB (2) and were initially available in WormBase, the database for *C. elegans* and related nematodes (3), before they shifted to computationally derived summaries. Computed summaries have the advantage that they are less labor-intensive, scalable and can be regularly updated by recomputing them from the latest database release (4–6). Indeed, the shift to computed summaries allowed WormBase to scale up from 6704 manually curated gene summaries for just one species to over 140 000 summaries for 9 species (R. Kishore, personal communication). Computationally derived summaries are also produced by FlyBase, the genetic database of *Drosophila melanogaster* (from here on Drosophila) (7) and the Alliance of Genome Resources (8). However, computed summaries are often difficult to read and in many cases fail to highlight the generally acknowledged function of the gene, for a couple of reasons.

Much of the data on gene function within databases is documented as Gene Ontology (GO) annotations (9,10). GO annotation has the advantage of using a controlled, hierarchical vocabulary that is shared between databases, making searching for all genes with a particular function more reliable and comparison between genes in different species easier. However, while paper-by-paper GO annotation can capture the many different roles of a gene product, the key function can be obscured within a large number of annotations. In addition, many of the early papers characterizing the main function of well-known genes had already been curated prior to the development of the GO in 1998 (10), meaning that high-quality annotations based on experimental evidence may be missing for the main function.

Another approach to generating gene summaries is the community curation initiatives utilized by the Human Gene Wiki (11) and the Mark2Cure project (12), but this relies on knowledgeable parties making the effort to contribute and, without some form of checking, can result in accumulation of erroneous information. FlyBase has also experimented with a Wiki approach, but there was little response from the community.

We sought an efficient way to provide accurate short summaries of Drosophila gene function for FlyBase, which ideally would also be accessible to a general biologist audience. Writing the summaries ourselves would be a very time-consuming approach: the Drosophila genome contains ~5000 protein-coding genes that merit a brief gene summary (see Materials and methods), and we have found that a curator requires at least an hour to research and summarize each gene’s function, meaning it would take over 3 years for a full-time curator to complete the project. Given our previous success in soliciting input from experts in the Drosophila research community to curate newly published research (13), we decided to try a similar approach to obtain gene summaries. Community submission of gene summaries required several problems to be addressed: (i) identification of genes sufficiently characterized to merit a summary, (ii) selection of the most knowledgeable researchers, (iii) mechanisms to contact experts and track responses, (iv) how to deal with a lack of response and (v) ensuring consistency in content and format across summaries. Here, we describe an efficient pipeline to produce short summaries, which we call ‘gene snapshots’, and present our progress to date.

## Materials and methods

### Gene categorization

For the pilot study, we ranked genes using a score incorporating the following data: number of publications linked to the gene, number of page views for the FlyBase gene report page (obtained from Google Analytics), number of GO terms, the presence of a human ortholog and the presence of OMIM (14) records for the human ortholog (15). The weighted formula used to select 85 top scoring genes for the first cycle was as follows:

totalScore = GO score + evidence score + number of FlyBase gene page views/10 + number of publications on gene + presence of ortholog^*^20 + presence of ortholog in OMIM^*^50 (see Table 1 for description of the GO and Evidence scores.)

**Table 1:** Detailed descriptions of the GO scores and Evidence score variables from the totalScore formula

**Variable**	**Description**
**GO score**	Sum of the scores given to every GO term associated to a gene, adding 1 point per GO term, with ROOT terms giving 0.5 points and no term giving 0 points.
**Evidence score**	200, 100 and 50 points if there is at least one biological process, molecular function and cellular component GO term, respectively.

For the second cycle, we categorized the genes based on the presence/absence of alleles with phenotypic information in the associated papers. We excluded unnamed genes that had no classical alleles with phenotypic effects annotated to them and targeted all the remaining genes for which we could identify an expert author with an accessible email address.

For the third cycle, we applied a more restrictive filter in order to identify genes with enough data to merit a summary. We grouped the 13 907 protein-coding genes annotated for Drosophila (FB2016_02) into three categories based on the amount and type of annotation available in FlyBase. This included the presence/absence of experimental and non-experimental GO data, alleles with phenotypes and information on whether the gene had an ortholog in humans or other non-arthropod metazoa (using ortholog data in FlyBase sourced from OrthoDB v9 (15). We then selected only those genes with experimental GO data and alleles with phenotype (4981 genes). These were the genes that we consider as well-characterized enough to merit a summary.

**Table 2:** Detailed descriptions of the variables used in the scoring function for ranking authors based on their contribution to the knowledge about a gene

**Variable**	**Description**
**10 year-recent**	Number of papers published by the author on the selected gene in the last 10 years
**mutagen-or-nature-of-lesion**	Number of papers published by the author describing new genetic reagents (allele or transgenic construct) for the selected gene
**gene-counts-500-limit**	Number of papers published by the author on the selected gene, where the paper is linked to less than 500 genes in total (to exclude high-throughput analyses)
**classical-alleles-with-pheno**	Number of papers published by the author on the selected gene, where the paper describes phenotypes of classical allele(s) of the selected gene
**expGO**	Number of papers published by the author on the selected gene including experimental evidence GO terms (Biological Process and Molecular Function aspects)
**expression-plus-antibody**	Number of papers published by the author on the selected gene including expression data and/or antibody information
**gene-source**	Number of papers published by the author on the selected gene including ‘gene source’ information (i.e. papers first characterizing a gene)

### Ranking authors based on their contribution to the knowledge about a gene

In the pilot study, we simply ranked authors based on number of publications on the gene. In the second cycle, we designed an algorithm to predict which authors are the most likely to provide information on the function of each gene. This algorithm takes as input a number of different sources of information from the FlyBase database and produces a single numerical score for each author that is associated to a gene. The scoring function applied to these data for each author for each gene is as follows:

(10 year-recent/10 + mutagen-or-nature-of-lesion/5)^*^(gene-counts-500-limit/5 + 3^*^ classical-alleles-with-pheno +10^*^expGO + 10^*^expression-plus-antibody + 10^*^ gene-source) (see Table 2 for a description of the individual variables.)

To select authors who have worked on a gene recently and generated reagents to assess its function, the first term of the function sets the score to zero if the researcher has not published on the gene during the last 10 years or has not described any genetic tool. We also excluded authors who have not been last author on a publication on the gene at least once, to favor more experienced authors.

After producing the ranked list of authors for each gene, some authors were manually filtered out for various reasons (e.g. if they were retired or deceased) and the corresponding genes were then assigned to the next highest ranked author. This growing list of authors to exclude was then incorporated into the ranking algorithm so as to automatically exclude them from subsequent cycles. Some *ad hoc* manual reassignment was done also for authors who were the predicted most suitable author for a particularly high number of genes, to avoid overloading them with requests.

### E-mail address extraction

Several approaches were used to obtain author e-mail addresses. First, a FlyBase internal list of e-mail addresses associated to publications was used (13). However, this list was incomplete and often out of date. Additional e-mails were extracted from PDFs stored internally by FlyBase. A script making use of the *pdftotext* tool from the *poppler* library (https://poppler.freedesktop.org) was used to extract text from the PDF files and capture e-mail addresses using regular expressions (https://github.com/FlyBase/email-extraction). For a given author, this produces a set of e-mail addresses contained in the publications to which the author contributed, but this set will typically also contain the addresses of other authors. To obtain the address most likely belonging to the given author, another regular expression search for addresses containing the last name of the author was performed across this set. The search was also restricted to publications not older than 5 years, to reduce the probability that the obtained address is obsolete. The results were manually inspected to ensure that the obtained address was likely correct. If no address could be identified through this method for a given author, a manual web search was performed.

### Bulk e-mail sending process

We used the *Mail Merge 4.6.1* add-on of the Thunderbird software (https://addons.mozilla.org/thunderbird/addon/mail-merge/) to send bulk e-mails, specifying files containing the information needed to customize each e-mail to the specific author and gene. This custom information consisted of (i) the gene name, (ii) the gene symbol, (iii) the author surname and (iv) a link to the personalized response form. In addition, we included other summary information that was available to assist the author, namely, (i) the summary of any FlyBase gene group of which the gene is a member (16) and (ii) the summary for the gene from The Interactive Fly (http://www.sdbonline.org/sites/fly/aimain/3a-dtest.htm). For authors who were prioritized as the top author for up to five genes, they were sent individual e-mails for each gene. For those authors expert on six or more genes, we sent a single e-mail asking for a summary on all of the genes, which included an attachment of a formatted spreadsheet file that the author could fill in (the file name and path of which was included in the Thunderbird customization file). These spreadsheet files were produced from text files using a combination of the Mac Automator software, Microsoft Excel macros and bash scripts.

### Tracking author e-mails and responses

Authors asked for up to five genes and more than five genes were given deadlines for replying of 1 and 4 weeks, respectively. After this period of time, reminders were sent to non-responders unless they had replied indicating that they were not able to do so. A second reminder was sent after an additional period of the same length. In some cases, customized reminders were sent to authors who indicated specific deadlines that suited them.

The response tracking was performed in two different ways, depending on the number of genes associated to a top author: for authors expert on five or fewer genes, all the snapshots received were stored using the form builder *Wufoo* (https://www.wufoo.com); for authors that were asked for snapshots on six or more genes, replies were kept track of locally using spreadsheets.

### Specifying format in database and web display

Snapshots are loaded into the FlyBase Chado database (17) via proforma files for each gene storing the free text snapshot, the date stamp and the name of the contributing author. Genes with insufficient information for a summary are also date-stamped to record when this was last reviewed.

## Results

### General strategy and its refinement

Our goal was to develop automated methods to identify researchers most likely to be knowledgeable about particular Drosophila genes and e-mail each expert requesting they draft a concise summary of the gene’s main function(s). The summary received would then be edited and displayed within FlyBase.

We have performed 3 cycles of this process. Following each cycle, we analyzed the feedback provided by authors, especially regarding the accuracy of our approach for selecting experts, and explored the features of genes for which we did not receive summaries. These analyses allowed us to refine the process and expand the number of gene snapshots produced in subsequent cycles, as described below.

### Pilot study

First, all protein coding genes were ranked on the basis of a number of data types stored in FlyBase, including publication numbers, GO annotations and orthology. Eighty-five top-scoring genes were selected to perform a small pilot study. We manually chose one or two authors per gene based on the number of papers published on the gene of interest. We redundantly sent the same genes to multiple authors and sent reminder emails to non-responders. As a result, summaries were obtained for 44 genes.

The pilot strategy was especially useful in improving the type of information requested for a gene snapshot. We initially asked authors to describe the main functions of the gene and provide key references. However, this approach yielded summaries with great variety in content. For example, some authors focused on phenotypes while others concentrated on detailed biochemical interactions. In addition, much of the data provided was better described elsewhere on the FlyBase gene report page (e.g. orthology, protein domains, expression). We also realized that finding key references was particularly time-consuming and hindered the return of snapshots. Therefore, we provided more specific instructions in the following cycles, requesting only ‘a couple of sentences/bullet points for the gene describing 1) how the protein functions, 2) what pathway it is in (if relevant), and 3) what are its main biological roles, preferably using terms suitable for a general, non-Drosophilist audience’.

**Table 3:** An example illustrating how, for a given gene, various values quantify how much each author has contributed to the information stored in FlyBase on a given gene

**Author name**	**Suggested email**	**LTP** **Papers**	**10y** **Papers**	**Clas. Al.** **w Pheno**	**GO** **exp**	**Exp** **+ AB**	**Mut +** **Nat. Les.**	**Gene** **Src.**	**Final score**
Author1	author1@...	24	8	12	1	0	5	0	**91**
Author2	author2@...	9	5	7	3	0	5	0	**79**
Author3	author3@...	12	4	7	3	0	5	0	**75**
Author4	author4@...	28	6	9	0	0	7	0	**65**
Author5	author5@...	14	0	10	1	0	7	0	**60**
Author6	author6@...	17	2	6	2	0	5	0	**50**
Author7	author7@...	15	5	7	3	0	1	0	**38**
Author8	author8@...	28	10	4	0	0	4	0	**32**
Author9	author9@...	12	7	3	1	0	3	0	**28**
Author10	author10@...	14	1	2	0	3	3	0	**27**

A final score is calculated as a function of these values, and the authors can be ranked on the basis of this score. See Materials and methods for descriptions of the column headings. LTP papers: low-throughput papers; 10y Papers: papers in the last 10 years; Clas. Al. w Pheno: classical alleles with phenotypes; GO exp: experimental evidence GO terms; Exp + AB: expression plus antibodies; Mut + Nat. Les.: mutagen plus nature of lesion; Gene Src.: gene source.

The pilot study used a manual strategy to organize and collate information: author e-mail addresses were retrieved from their lab web pages, and e-mails were produced and sent individually; authors’ summaries were then received by e-mail and manually transferred to a spreadsheet. Although this approach was feasible with low numbers of e-mail recipients, it was clear that we would have to automate these processing steps to deal with larger numbers of genes and authors.

### Cycle 2—procedure for selecting experts on each gene

In the pilot cycle, we manually selected authors based simply on the number of their publications that were linked to the gene in FlyBase. While this criterion worked well for many genes, for others it did not. Some authors indicated that while the gene for which a summary was requested was associated with a large number of their publications, none of these were focused on characterizing that gene; instead, the gene was being used for another purpose, such as a genetic marker, or transgenic reporter. Other authors reported that they had not worked on the gene for many years or were not the main (first or last) author of a paper describing the gene’s function.

As manual selection of authors would be too labor-intensive to scale up to all genes, we developed a computational approach to assign an ‘expert score’ to every author who has published a paper on a given gene (see Materials and methods for details). Briefly, we counted the number of papers associated with each gene per author (‘last authors’ only) in FlyBase, noting how many of those papers were within the last 10 years to increase the chance of identifying authors still working on the gene. Information on the data present within each publication was also collected, with the aim of identifying authors working specifically on a gene rather than publishing genome-wide analyses. The following types of data in FlyBase were retrieved from each publication, representing experimental work characterizing gene function (Table 2): (i) phenotypes of classical mutant alleles, (ii) GO annotations based on experimental evidence, (iii) gene expression or generation of an antibody against the gene product, (iv) generation or characterization of mutant alleles of the gene and (v) data associated with naming the gene.

We collated this information for each author from all their papers, so that each author was linked to a set of values related to their publications on a gene (Table 3). We then tested various ways of weighting this information to calculate the expert score for each author per gene, evaluating the success of each scheme by examining 20 particularly well characterized genes. The most effective scheme assigned high scores to publications that describe mutant alleles and their phenotypes or experimentally supported GO annotations, and low scores to publications that reported on the gene as part of high-throughput experiments and screens (identified as publications that report data on a very large number of different genes). The final weighting scheme also required that authors had published on the gene in the last 10 years or contributed to a publication describing a mutant or other genetic construct for the gene.

As noted above, we also needed to automate the acquisition and processing of emails (see Materials and methods for details). We computationally retrieved e-mail addresses from recently published articles where the gene expert appeared as last author. When sending e-mails, we designed a template with custom information, allowing us to tailor the content of each e-mail to each author. The template was used by e-mail software to send bulk e-mails to hundreds of researchers. To enable collation of summaries, we set up an online form using Wufoo’s cloud storage database (https://www.wufoo.com) so that each e-mail to an author included a link to a form pre-filled with the gene name and the author’s name.

With these improvements in hand, we performed a second cycle of requests for gene summaries. We applied the algorithm to all 13 907 Drosophila protein coding genes annotated in FlyBase at the start of this project (FB2016_02) and identified an expert author with an accessible e-mail address for ~4000 genes. When more than five gene summaries were requested from the same author, a spreadsheet was attached to a single email including all the genes—we surmised that this approach was more likely to elicit a response than sending six or more separate e-mails. All authors were e-mailed once and sent a reminder after 2 weeks. When the effect of sending reminder e-mails was analyzed, we found that the first reminder resulted in substantial numbers of snapshot submissions but, in line with results from a similar effort involving direct e-mailing of authors (13), the response to the second reminder was low (Table 4). If there was no response from the second reminder, we contacted the second top author. The response rate from this author was in line with the results from the first round of e-mails and substantially higher than the second reminder to the first-ranked author. Based on these results, we decided not to send a second reminder to the first ranked author in the third cycle.

**Table 4:** The effect of reminders on the fraction of genes for which snapshots were obtained

**Cycle**	**Initial e-mails**	**1st reminder**	**2nd reminder**	**2nd ranked author**
Second cycle (1690 genes)	496/1690 (29%)	355/1104 (32%)	76/672 (11%)	205/693 (30%)
Third cycle (692 genes)	160/692 (23%)	128/532 (24%)	NA	NA

Note: responses are shown only for authors assigned up to five genes. Total number of genes are followed by percentages in parentheses, and all percentages are relative to the number of genes falling into the given category

**Figure 1:**
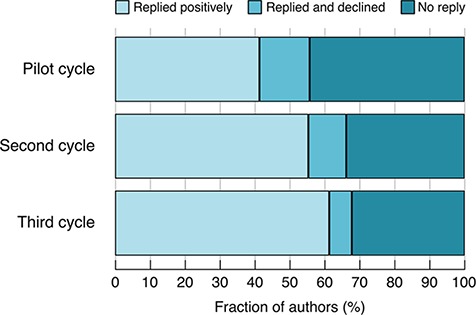
Author response rates in the different cycles. Pilot cycle: semi-manually selected authors. Second cycle: predicted authors, no gene categorization. Third cycle: predicted authors, with gene categorization.

This second cycle of emails resulted in authors making ~1800 submissions, corresponding to 1580 unique snapshots. (Some authors gave us additional, unsolicited, snapshots that overlapped with ones already received.) This represents 11% of all protein-coding genes and 33% of the genes for which e-mails were sent. The improvements made within Cycle 2 resulted in an increased fraction of authors contributing a snapshot and a reduction in the fraction of non-responders (Figure 1).

### Cycle 3—measuring the amount of data linked to the function of each gene.

Despite our efforts to improve the author selection algorithm, we still received many replies from authors stating they did not know enough about the gene to write a summary or that the gene was essentially uncharacterized. This suggested that we should evaluate more carefully whether enough was known about a gene to merit requesting a gene snapshot. To assess this, we examined how the response rate in Cycle 2 related to the amount of data on the gene in FlyBase, quantifying (i) GO annotations, divided into those based on experimental evidence, other evidence and no data, (ii) the presence/absence of classical mutant alleles with associated phenotype data and (iii) orthology to genes in other organisms (Materials and methods; Table 5). The highest frequency of response correlated with the presence of experimentally evidenced GO annotation data.

**Table 5:** The rate of snapshot submission in the second e-mailing cycle, with genes stratified by the type of annotation data in FlyBase

**Gene category**			**# genes**	**# genes e-mailed**	**# snapshots received**	**Snapshots received/e-mailed**	**Snapshots received/total**
GO data?	Alleles?	Orthologs?					
**Y (exp)**	Y	Y	3214	2578	937	**36%**	**29%**
**Y (exp)**	Y	N	813	635	260	**41%**	**32%**
**Y (exp)**	N	Y	1573	491	144	**29%**	**9%**
**Y (exp)**	N	N	601	213	114	**54%**	**19%**
Y (non-exp)	Y	Y	758	264	37	14%	5%
Y (non-exp)	Y	N	196	61	11	18%	6%
Y (non-exp)	N	Y	2544	271	43	16%	2%
Y (non-exp)	N	N	923	110	25	23%	3%
no GO	Y	Y	155	42	1	2%	1%
no GO	Y	N	272	48	4	8%	1%
no GO	N	Y	517	22	1	5%	0.2%
no GO	N	N	2341	40	3	8%	0.1%
Total			13 907	4775	1580	33%	11%

Abbreviations: exp = experimental GO data; non-exp = non-experimental GO data; no GO = no GO data available; Alleles = Alleles with phenotypes

Based on these analyses, we identified 4981 genes that have experimental GO annotations and associated alleles with phenotype annotations—we define these as sufficiently well-characterized to merit a summary. We then carried out a third cycle of e-mail requests, requesting information on 692 genes within this set for which we had not yet received a snapshot, and receiving information on 288 (Table 4). The improvements made in the third cycle further reduced the fraction of authors declining to write a snapshot and further increased the percentage of positive responses, compared to previous cycles (Figure 1).

**Figure 2:**
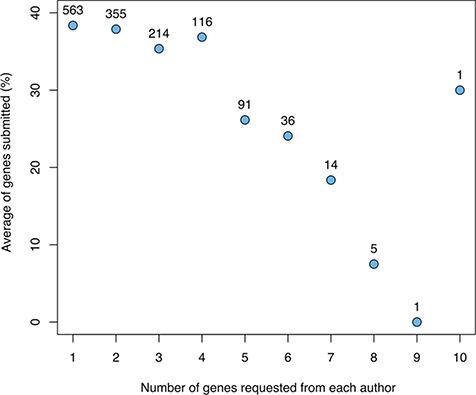
The relationship between the number of genes for which a given author was asked to provide snapshots and the average fraction of genes for which snapshots were returned (not including authors sent a spreadsheet with many genes). The numbers above the data points indicate the number of authors in each category.

Aggregating the results across cycles, we studied the relationship between the number of genes for which a given author was asked to write a snapshot and the number of snapshots returned (Figure 2). This shows a clear negative correlation, with a substantial drop-off when requesting more than four snapshots.

### Editing and display of snapshots

Submitted gene summaries were revised for length and consistency. An average of 200–250 snapshots per week were produced during early stages of the project. The summaries were proofread by a second curator before being entered into the FlyBase database. The first gene snapshots went live on the FlyBase website in June 2016 (release FB2016_04). At that time, we used a variety of approaches to highlight them, including a commentary on the homepage, a page thanking those who had contributed snapshots and a YouTube video (https://www.youtube.com/watch?v=6IDlJGXdIP8).

**Figure 3:**
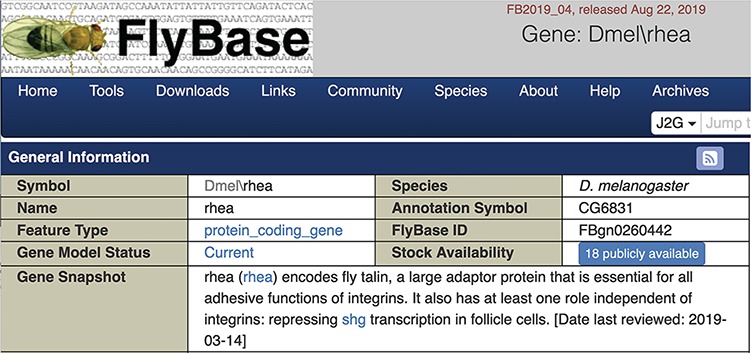
Screenshot of the top of a gene report page showing the Gene Snapshot section.

We chose to display the gene snapshots within the uppermost ‘General Information’ section of FlyBase gene reports (Figure 3). The date the snapshot was added or last reviewed is also shown. For genes currently lacking a snapshot, a link is given inviting contributions. Specific gene snapshots can also be downloaded in bulk via the Batch Download tool and the entire set of snapshots is available as a precomputed file.

At the time of writing (FB2019_04), there are snapshots for 3123 genes, representing 62% of our target of the ~5000 protein-coding genes identified as having sufficient associated data to merit a snapshot. (Note that this figure includes snapshots from additional rounds of the final method (Cycle 3) and 86 unsolicited summaries that were submitted by researchers via the website.) Future plans to attain our targets are discussed below.

## Discussion

**Figure 4:**
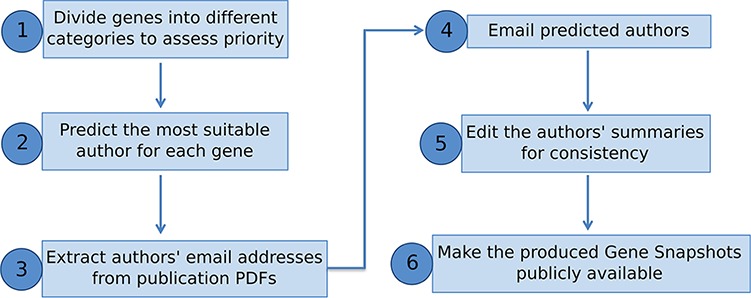
Overview of pipeline to produce Gene Snapshots.

We have presented a pipeline to produce manually written gene summaries in bulk by soliciting expert knowledge from the research community (Figure 4). In summary, curated data stored in a genetic database, like FlyBase, can be used to identify genes meriting a summary and to generate an expertise profile of researchers. Experts on any given gene can then be emailed directly and asked to provide a few key points on that gene’s function. The received information is then edited for consistency and included within the database/website for the benefit of the whole research community.

The application of this approach within FlyBase has been very successful, generating 1800 gene snapshot submissions within a 3-month period and a total of 3123 snapshots to date. Besides matching the right researcher to the right gene(s), we believe there are two key reasons for this success. First, directly emailing authors with clear, brief instructions of the information required is hugely beneficial, especially compared to a general request that the community contribute. We have seen evidence of this before, both in the positive response in a previous related project (13) and in a failed attempt to acquire gene summaries via a FlyBase community gene wiki. Further evidence is provided by the fact that only 86 unsolicited snapshots have been submitted to FlyBase. The second reason is the willingness of >1000 Drosophila researchers to prepare draft gene summaries, reflecting the long-standing collaborative nature of this community. Additional reasons for the positive outcome of this project may include the high visibility of the resulting snapshot on FlyBase gene reports (Figure 3) and the acknowledgement of all contributors on a dedicated web page (https://wiki.flybase.org/wiki/FlyBase:Gene_Snapshot_Contributors).

Gene snapshots have proved to be a valuable addition for researchers. Prior to this project, the acknowledged key function of many well-characterized Drosophila genes was not clearly or concisely shown within FlyBase, either on individual report pages or available in bulk file. For example, a well-studied gene such as *decapentaplegic* can be associated with 100 or more different GO terms, but these may not readily indicate the primary function of the gene product. Gene snapshots have succeeded in solving this issue, as judged by the many positive anecdotal comments we have received and the fact that gene snapshots were ranked as the top ‘recent addition’ to the database and the ‘best available summary’ in recent surveys of the FlyBase community. Moreover, we have received suggested corrections for <10 gene snapshots to date, suggesting that users generally find that they are accurate and informative. FlyBase snapshots also now appear on the Drosophila gene reports within the Alliance of Genome Resources database (8), which integrates data from several model organisms into a central resource.

Several problems were encountered throughout the course of this project—these were addressed and solved through iterative development cycles. For example, a number of authors declined our request because they no longer work on Drosophila or had stopped studying the gene in question. We had attempted to screen these out by making sure the author had published on the gene within the last 10 years, but some cases still slipped through, often because the last author that we contacted was not in fact the corresponding author for the recent work. Our pipeline also included top-ranked authors who were no longer active, which could be avoided if we had an up-to-date database of *Drosophila* researchers. Another issue was that some e-mail addresses extracted from publications were no longer in use, highlighting the value of systems for permanent researcher identification such as ORCID (18). Other researchers replied to our request for snapshots saying they could not contribute because they were too busy. While it is difficult to be certain, we suspect that these same issues also explain why many researchers did not respond at all to our emails. In many cases, we were still able to obtain a snapshot by contacting the author ranked as the second expert on a given gene (Table 4).

Data on new mutant alleles, expression of the gene product and GO annotation inferred from mutant phenotypes all contribute to the estimation of the extent to which a gene has been characterized and the papers describing that characterization. A by-product of this effort is that we have been able to use a similar approach to identify which specific papers describe relevant work on each gene and highlight these in FlyBase gene reports as ‘representative publications’ (7, to be described in detail elsewhere). Highlighting such papers is particularly useful for genes with very long reference lists.

We continue to seek new ways to encourage authors to contribute, without overly pestering them. As mentioned above, FlyBase gene reports currently lacking a snapshot contain a link inviting researchers to make a direct submission. Furthermore, we are updating the FlyBase Fast-Track Your Paper tool, which allows authors to carry out a light curation of their recent publications (13), to allow the submission of gene snapshots as part of that process. Inevitably, there will remain a subset of well-characterized genes that merit a summary but for which we have not received a submission from the research community, despite repeated efforts. We plan to write our own snapshots for the best characterized of these genes, based on a review of the data housed in FlyBase and key literature references.
